# A practice of using five-colour chart to guide the control of COVID-19 and resumption of work in Zhejiang Province, China

**DOI:** 10.1038/s41598-021-90808-0

**Published:** 2021-05-31

**Authors:** Fan He, Xiaopeng Shang, Feng Ling, Zhiping Chen, Tiehong Fu, Junfen Lin, Zhen Wang

**Affiliations:** 1 Zhejiang Provincial Center for Disease Control and Prevention, Zhejiang, China; 2 Zhejiang Provincial Health Commission, Zhejiang, China

**Keywords:** Viral infection, Preventive medicine, Epidemiology

## Abstract

Since the outbreak of COVID-19 in December 2019 in Wuhan, Zhejiang has become the province with the largest number of cases. The aim of this article is to present Zhejiang province’s experience of establishing an accurate and smart control mechanism for epidemic prevention and control and resumption of work and production using a ‘five-colour epidemic chart’. The number of confirmed cases, proportion of local cases, and occurrence of clustered outbreaks were used as evaluation indicators to calculate the county-level epidemic risk and were assigned different weight coefficients; the absence of cases for 3 and 7 consecutive days was used as the adjustment index. When the first chart was published on February 9, there were 1 very-high-risk, 12 high-risk, and 12 low-risk counties. Under the five-colour chart, Zhejiang began to adopt precise measures to prevent and control the epidemic and resume work and production. By February 24, the low-risk counties had expanded to 82, with no high-risk and very-high-risk counties. The epidemic situation in Zhejiang province has been effectively controlled. The experience of epidemic prevention and control in Zhejiang is worthy to be emulated and learned by other countries and regions.

## Introduction

In December 2019, a novel coronavirus was found to cause human illness in Wuhan, Hubei province, China^1–3^. It can cause fever, dry cough, and fatigue in patients. It can also cause the elderly, people with chronic underlying diseases, late-pregnancy and perinatal women, and obese people to be critically ill patients, most of them have breathing difficulties and hypoxemia one week after the onset, severe cases may progress to acute respiratory distress syndrome, septic shock, difficult to correct metabolic acidosis, coagulation dysfunction, and multiple organ failure, etc. Symptoms in children are relatively mild. A very small number of children may have multiple system inflammatory syndrome (MIS-C), similar to Kawasaki disease or atypical Kawasaki disease, toxic shock syndrome or macrophage activation syndrome, etc., which mostly occur in the recovery period^4–7^. Just one month later, on January 28, 2020, the virus spread to 31 provinces (autonomous regions and municipalities) across the country. As of April 13, 2021, a total of 90,447confirmed cases and 4,636 deaths were reported across the country^8^. On 11 February 2020, the novel coronavirus disease was named COVID-19 by the World Health Organization (WHO)^9^, and the virus causing COVID-19 was named SARS-CoV-2 by the International Committee on Taxonomy of Viruses^10^.

Zhejiang Province, located in eastern China (longitude 118°24 to 122°26 E; latitude 27°31 to 31°10 N), consists of 11 prefectures and 90 counties and has a population of more than 50 million. Geographically, Zhejiang province borders Shanghai, Jiangsu, Anhui, Jiangxi, and Fujian provinces. It was estimated that before the Spring Festival, a traditional Chinese festival, more than 200,000 people returned to Zhejiang from Wuhan of Hubei province. Since the first case was imported from Wuhan on 20 January, Zhejiang has quickly become the province with the largest number of cases except Hubei province^11,12^. However, the Zhejiang government initiated the level I response to major public health emergencies on 23 January in response to the epidemic, and most prefecture-level cities implemented the most stringent measures^13^. For example, Hangzhou municipal people’s government issued a circular on the implementation of 10 measures on 4 February^14^; the Jiaxing Municipal Disease Prevention and Control Leadership Group issued seven orders to control the epidemic on January 30^15^ and used smart methods to control the spread of the epidemic in 33 days (since February 22, no new confirmed cases were reported in Zhejiang province). In all those measures, the ‘one chart, one code and one index’ method is the most effective; the ‘one chart’ (five-colour epidemic chart) has especially become the guide for the prevention and control of the epidemic and the resumption of work and production, and has been widely used in the country. This study aimed to introduce the composition and practice of the indicators of the five-colour epidemic chart and to provide a reference for other countries and regions to combat the COVID-19 epidemic.

## Results

### Epidemiological characteristics of the indicators for risk assessment

**Figure 1:**
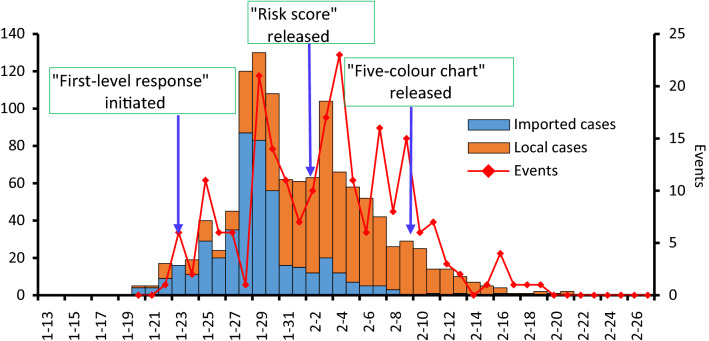
Timeline of the epidemic situation and action for COVID-19 in Zhejiang province of China.

Figure 1 shows the COVID-19 epidemic curve with the number of confirmed cases plotted by date of diagnosis from 13 January to 27 February 2020 and the change curve of the number of clustered outbreaks. Imported cases and local cases are stacked to show total daily cases by date of diagnosis.

On 20 January 2020, Zhejiang reported the first COVID-19 case coming from Hubei province. As of 27 February, 1205 confirmed COVID-19 cases were reported. Epidemic curves indicate that daily newly confirmed cases increased rapidly from 20 to 31 January. The peak diagnosis date for all cases occurred on January 29, with a maximum of 132 cases reported on that day. Subsequently, except for the unusual increase in the number of cases on February 3, the number of daily reported cases gradually decreased. From 17 to 22 February, the number of confirmed cases per day was less than two, and there were no confirmed cases reported from 22 to 27 February.

From the epidemic curve, we can see that from 20 to 30 January, the proportion of imported cases among newly diagnosed cases was greater than that of local cases. Since 31 January, the daily newly confirmed cases were mainly local cases.

From 20 January to 27 February, a total of 218 cluster COVID-19 outbreaks were reported in Zhejiang province. These outbreaks were mainly concentrated from 19 January to 9 February, with an average of 13 outbreaks per day. The highest number of cluster outbreaks was reported on 4 February, when 23 outbreaks were reported. Among the 218 clustered outbreaks, 200 were family clustered outbreaks, accounting for 91.74%; 18 were non-family clustered outbreaks, accounting for 8.26%. A total of 917 confirmed cases were reported in all clustered outbreaks, with the highest one reporting 55 cases.

### Risk assessment of COVID-19 by counties

**Figure 2:**
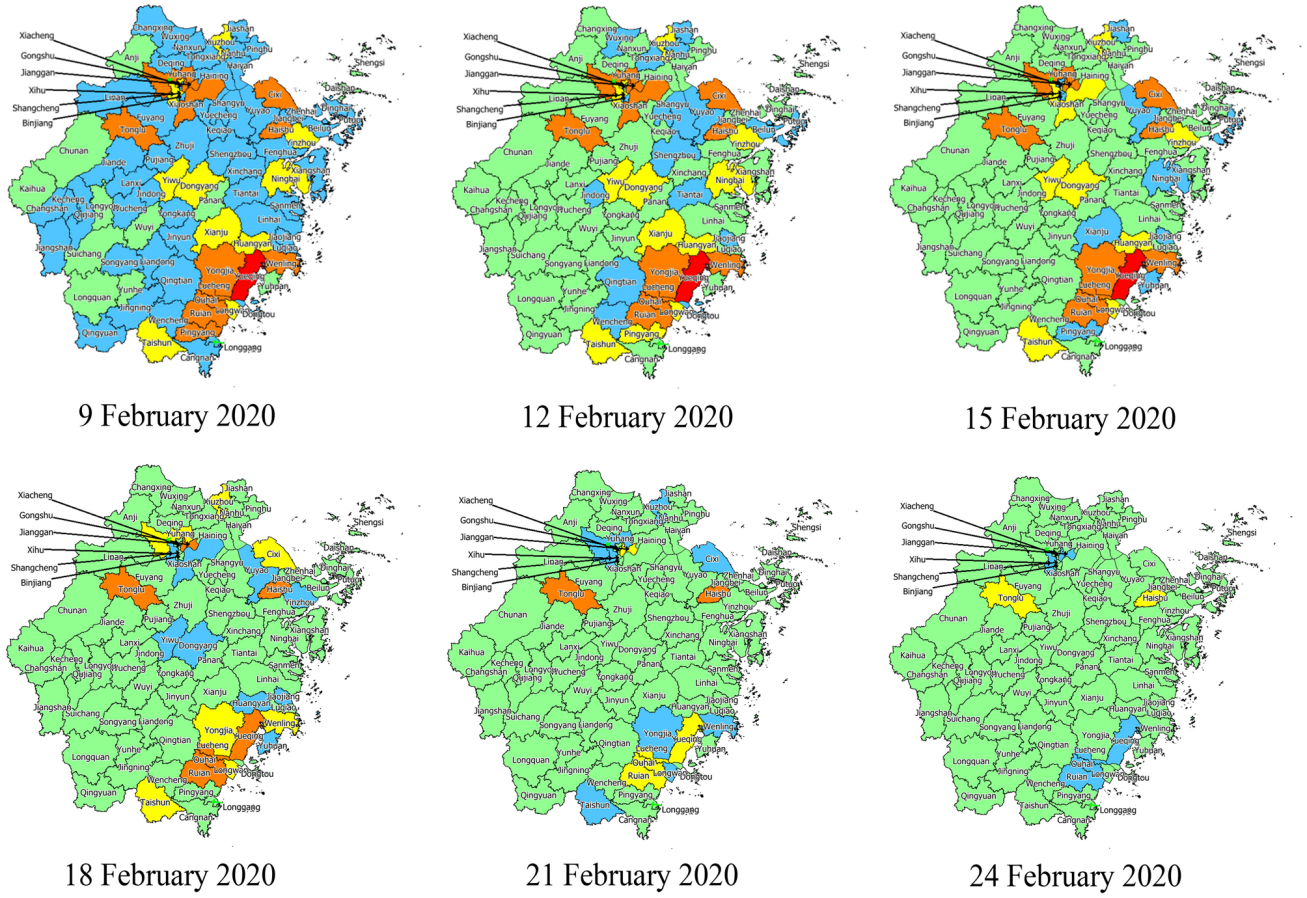
Five-colour epidemic charts of county-level epidemic risk in Zhejiang province from 9 to 24 February, 2020. Statement: The base map was provided by the Department of Natural Resources of Zhejiang Provincial (Map approval number: 浙S(2020)17). We used the MapInfo software (V16.0 Chinese; URL link: https://www.precisely.com/product/precisely-mapinfo/mapinfo-pro) to create the maps.

**Figure 3:**
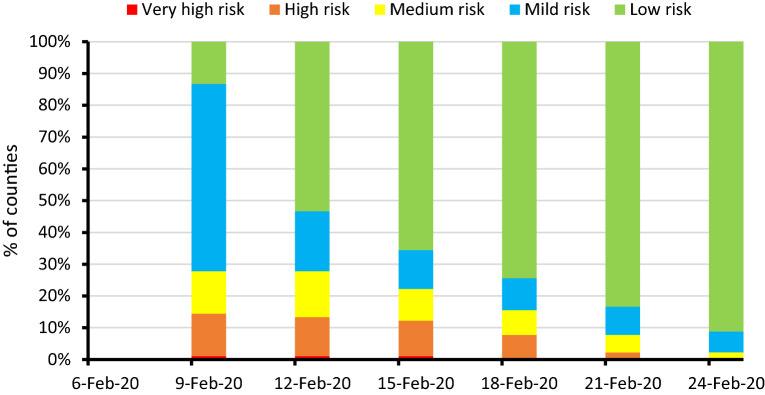
Trends in the proportion of regions with different epidemic risk levels in Zhejiang province from 9 to 24 February, 2020.

On 9 February, we calculated the county-level epidemic risk index according to the number of confirmed cases, proportion of local cases, number of clustered outbreaks, and number of confirmed cases reported in clustered outbreaks of each county as of 8 February, as well as the location where no new cases were reported for several consecutive days. Among the 90 counties in Zhejiang province, Yueqing had the highest epidemic risk score of 95. The 12 counties that never reported any cases had an epidemic risk score of 0. According to the risk classification rules mentioned in the method section, there were 1 very-high-risk, 12 high-risk, 12 medium-risk, 53 mild-risk, and 12 low-risk counties. According to the adjustment rule, the county epidemic risk level was adjusted every three days. As the number of confirmed cases reported daily decreased, the epidemic risk level gradually decreased, and the proportion of low-risk areas gradually increased (Figs. 2, 3). By 24 February, there were no very-high-risk and high-risk counties, 2 medium-risk counties, 6 mild-risk counties, and 82 low-risk counties.

## Discussion

The five-colour epidemic chart was produced for scientific guidance to resume work and production when the epidemic situation in Zhejiang province gradually slowed, and the number of confirmed cases reported daily gradually decreased. It has an important reference value and guiding significance. Here, it is necessary to explain the principles and assessment basis of the five-colour epidemic chart: (1) The basis of the assessment is the authenticity, reliability, and timeliness of the data. (2) According to the different stages of provincial epidemic development, relevant indicators were selected based on the epidemiological characteristics of COVID-19 transmission and the main factors affecting its spread. In this study, the experts selected local cases and the situation of the cluster outbreaks as indicators because Zhejiang province had coexisting imported and local cases, and the imported epidemic gradually shifted to local transmission. The risk of local transmission needs to be strictly managed to truly control the epidemic. At the same time, experts assigned different weights to the risk of transmission of cluster outbreaks within and outside the family. (3) A comprehensive assessment was conducted based on the composition of cases in the county (the number of imported and local cases) as well as the number of new cases and the cluster outbreaks in the county within an average incubation period (seven days)^5,16–17^. (4) The method of calculation of county-level epidemic risk is simple and clear, and the five-colour epidemic chart that displays the assessment results are intuitive and visual, which is highly practical, easy to operate, and replicable. (5) The assessment should be carried out dynamically according to the actual local situation and the epidemic stage of the disease, and the assessment indicators and their weights should be adjusted appropriately when necessary to scientifically correct the assessment results.

The five-colour epidemic chart has been widely used since its publication^18–21^. Similar to a ‘barometer’ for the prevention and control of the COVID-19 epidemic, the five-colour epidemic chart is conducive to enhancing the public’s awareness of self-protection and is also an important basis for classified strategies for resumption of work and production^22^. On 9 February, Zhejiang province announced resumption of work and production by region, time section, and industry following the principles of ‘offering classified guidance according to time sections and local conditions’. Work and production was resumed by five regions specified in the ‘five-colour epidemic chart’. Counties with a medium epidemic risk should insist on prioritising epidemic prevention and control, and promoting work and resuming production in a safe and orderly manner. Counties with a low and mild epidemic risk should fervently promote work resumption under the precondition of safety and controllability, while high-risk countries should continue to be fully committed to doing a good job in epidemic prevention and control^23^.

Precise and differentiated epidemic control strategies were adopted based on the risk level corresponding to the five-colour epidemic chart in each county to combat the epidemic. According to the five-colour epidemic chart, corresponding five-colour chart of work resumption rate, five-colour chart of transportation, and five-colour chart of logistics were launched, making each work more focused on classification and accuracy, and effectively improving the overall performance. Based on the five-colour epidemic chart combined with big data analysis and artificial intelligence technology, health code and control and smoothness indices came into being, which together constitute the core of the current precision intelligent control mechanism in Zhejiang province – ‘one chart, one code, and one index’^24^. The five-colour epidemic chart, health code, control, and smoothness indices provide effective channels for governments at different levels to classify prevention and control efforts and facilitate the production enterprises and lives of people. These measures have provided a decision-making basis for actively seeking, identifying, and scientifically responding to changes, so that an accurate and scientific victory can be won in the battle against the epidemic and in the economic and social development^25^.

Under the guidance of the five-colour epidemic chart, orderly resumption of work and production was carried out in all regions of Zhejiang province. As of 27 February, with the exception of mild-risk areas, full resumption of work was achieved, and the recovery rate of production capacity reached over 75%. There were also no new COVID-19 cases reported among more than 500,000 returning workers from outside the province. The ‘five-colour epidemic chart’ will continue to indicate 90 counties of the risk of spreading the disease within the county in the next stage and will serve as an important basis for formulating prevention and control measures and policies related to resumption of production and work.

As the current global epidemic continues^26^, other countries and regions are facing issues on classifying and accurately implementing strategies for regional epidemic prevention and control, and quickly and effectively restoring production and life in the later stages of epidemic prevention and control; the five-colour epidemic chart is undoubtedly a method worth learning from.

## Limitation

This is a significant study conducted in the early stages of the pandemic and at a time when the prevention and control situation is very critical. Under the guidance of the five-colour chart, the COVID-19 epidemic in Zhejiang Province has been effectively controlled, and people's production and life have been restored in an orderly manner. But at the same time, we also see that our research has some limitations. One of them is that we use qualitative methods, including Delphi method and expert consultation method^27^, to determine county-level epidemic risk indicators and their weights. Furthermore, the indicators we use to evaluate the risk of the county epidemic are too simple and incomplete. In addition, the use and application of the five-colour chart has obvious local characteristics. In other places, the indicators and their weights should be adjusted appropriately according to the characteristics of the local epidemic. In further research, we will adopt quantitative or semi-quantitative assessment methods to construct a complete index system of county-level epidemic risk assessment, making it more scientific, rigorous and universal.

## Methods

### Study design

According to organising 15 experts in epidemiology, statistics, and health management to conduct sufficient discussion, and combined with the actual situation in Zhejiang province, we choose the Delphi expert consultation method to determine the county-level epidemic risk assessment index, and use the expert consultation method to determine the index weight and the subsequent adjustment rules. We selected the total number of confirmed cases, the proportion of local cases, and the clustered outbreaks (non-family and family clustered outbreaks) as positive indicators, accounting for 50%, 30%, and 20%, respectively, to calculate the county-level epidemic risk. Meanwhile, the absence of newly confirmed cases for three consecutive days (or seven days) was selected as the negative indicator, which was used to adjust the county-level epidemic risk score.

In the initial stage of assessment, the total county-level epidemic risk score ($S_{i}$) = score of total confirmed cases ($\alpha_{i}$) + score of local case proportion ($\beta_{i}$) + clustered outbreak score ($\gamma_{i}$) ($i$ = 1,2,3,4…90).where $\alpha_{i}$ is based on the number of confirmed cases on the day before the assessment; the county with the highest number of cases was given a full score of 50, and the rest of the counties’ score was calculated by the proportion of the number of confirmed cases.

$\beta_{i}$ is based on the data on the day before the assessment; the county with the highest proportion of local cases to the total number of confirmed cases was given a full score of 30, and the proportions of local cases in other counties were calculated proportionally.

$\gamma_{i}$ refers to the number of clustered outbreaks and the total number of cases in clustered outbreaks, each accounting for 50%. Among them, the clustered outbreaks were divided into 30% of family clustered outbreaks (A1) and 70% of non-family clustered outbreaks (A2). The number of cases was divided into family cluster cases (B1) accounting for 30% and non-family cluster cases (B2) accounting for 70%. The total clustered outbreak score = (0.3 A1 + 0.7 A2) × 0.5 + (0.3 B1 + 0.7 B2) × 0.5. We assigned the highest clustered outbreak score in the county to a full score of 20 and calculated the score of the clustered outbreaks in other counties proportionally.

Then we used the negative indicator to adjust the county-level epidemic risk score. The rule was that if there were no case reports for three consecutive days, 5% was deducted from the total score, and if no case reports for seven consecutive days, 10% was deducted from the total score.

According to the adjusted $S_{i}$, the epidemic risk level of each county was classified as follows: ≧ 90 points, very-high-risk areas; ≧ 60 and < 89 points, high-risk areas; ≧ 20 and < 60 points, medium-risk areas; ≧ 10 and < 20 points, medium-risk areas; < 10 points, low-risk areas.

Thematic maps are represented by red, orange, yellow, blue, and green colours, respectively, i.e., a five-colour epidemic chart^28^.

The county-level epidemic risk was adjusted every three days according to the subsequent adjustment rules. The rules for increasing risk levels were as follows: the risk was increased by one level if the total number of newly confirmed cases for three consecutive days in the five days before the assessment was 10 or more, or there was report of one non-family cluster outbreak with the number of cases greater than or equal to 5, or two or more non-family cluster outbreaks within three days before the assessment. In areas that were initially at low risk, as soon as cases were reported within three days before the assessment, it was raised to mild risk. The risk level reduction rule was as follows: if no case was reported for six consecutive days before assessment, the risk was reduced by one level.

### Data source

According to the latest version of the guideline on the diagnosis and treatment of novel coronavirus pneumonia released by the National Health Commission^5^, a confirmed case was defined as a suspected case with respiratory specimens that tested positive for the COVID-19 by real-time reverse-transcription polymerase chain reaction (RT-PCR) or a genetic sequence that is highly homologous with the known novel coronavirus. A cluster outbreak refers to two or more confirmed cases or asymptomatic infections that are found in a small area (such as a family, a construction site, a unit, etc.) within 14 days, with the possibility of human-to-human transmission due to close contact, or a possibility of infection due to co-exposure.

As a legal infectious disease, Chinese law required all COVID-19 cases to be immediately reported to China’s Infectious Disease Information System. Demographic information, consultation information, and epidemiological information related to each case were investigated and collected by local epidemiologists and public health workers and transmitted to the information system. Based primarily on epidemiological investigations, cases were categorised as imported cases if they had resided in or visited Wuhan and surrounding areas or other communities with cases reported within 14 days before the onset of illness, or if they had close contact with someone with fever or respiratory symptoms. Otherwise, it was a local case. According to whether the epidemic outbreaks occurred in the same family, clustered outbreaks were divided into family and non-family clustered outbreaks.

In this study, data of all 1205 COVID-19 cases reported and 218 clustered outbreaks reported in Zhejiang province were extracted from China’s Infectious Disease Information System by the end of 27 February 2020.

### Statistical analysis

The epidemic curve for confirmed cases was constructed based on the date of diagnosis, and key dates relating to control measures were overlaid to aid interpretation. The dates of diagnosis for imported cases and local cases were stacked to show total cases over time. The curve for clustered outbreaks was also overlaid with the number of cases versus the date of diagnosis. MapInfo Pro (V16.0 Chinese ; Pitney Bowes Software Inc., Stamford, CT, USA) was used to map the geographic distribution and variation.

### Ethics approval

This study was in response to a public health emergency. As such, it was exempt the Zhejiang Provincial Center for Disease Control and Prevention’s requirement for ethical approval and informed consent. All methods were carried out in accordance with relevant guidelines and regulations.
